# Supplementary material for: Soil warming during winter period enhanced soil N and P availability and leaching in alpine grasslands: A transplant study
Source: PLoS One. 2022 Aug 2;17(8):e0272143. doi: 10.1371/journal.pone.0272143 (PMC9345486; doi:10.1371/journal.pone.0272143)
Supplement: S1 Table — Data were obtained during the destructive sampling in September 2015, 2 years after exposure of the transplants to the field conditions. The data were used to calculate means and standard deviations given in the paper. (DOCX) [file pone.0272143.s001.docx]

**Table S1. Raw data characterizing soil and plant chemistry a soil biochemistry in transplants left in site and mowed downward in two valleys in the Tatra Mountains.** Data were obtained during the destructive sampling in September 2015, 2 years after exposure of the transplants to the field conditions. The data were used to calculate means and standard deviations given in the paper.

| **origin** | pH | Soil N  (mg g^-1^) | Soil C  (mg g^-1^) | Dissolved organic C (mg kg^-1^) | Dissolved N (mg kg^-1^) | Soluble reactive P (μg kg^-1^) | Dissolved NH4-N  (μg kg^-1^) | Dissolved NO3-N  (μg kg^-1^) |
| --- | --- | --- | --- | --- | --- | --- | --- | --- |
| **FU-H** | 4.35 | 8.9 | 123.8 | 96.3 | 8.1 | 205 | 1353 | 4364 |
| **FU-H** | 4.12 | 6.6 | 84.4 | 91.9 | 5.4 | 339 | 1516 | 2913 |
| **FU-H** | 4.75 | 4.5 | 60.7 | 98.3 | 5.7 | 228 | 529 | 1631 |
| **FU-H→L** | 5.17 | 8.0 | 122.4 | 167.8 | 53.7 | 345 | 4446 | 3431 |
| **FU-H→L** | 4.39 | 8.6 | 112.7 | 137.2 | 49.4 | 304 | 1329 | 5448 |
| **FU-H→L** | 4.39 | 7.2 | 97.8 | 101.0 | 28.2 | 420 | 2163 | 8019 |
| **FU-L** | 4.82 | 7.0 | 99.6 | 67.8 | 3.4 | 382 | 829 | 839 |
| **FU-L** | 4.6 | 8.1 | 107.7 | 91.2 | 5.7 | 421 | 1130 | 958 |
| **FU-L** | 4.71 | 7.5 | 103.7 | 114.5 | 7.9 | 460 | 1431 | 1076 |
| **VS-H** | 4.69 | 4.9 | 67.0 | 55.1 | 2.6 | 77 | 546 | 1116 |
| **VS-H** | 5.03 | 2.4 | 36.1 | 29.3 | 3.1 | 60 | 456 | 2650 |
| **VS-H** | 4.74 | 2.2 | 33.2 | 40.5 | 5.3 | 75 | 767 | 3347 |
| **VS-H→L** | 4.64 | 1.8 | 33.9 | 56.7 | 4.1 | 85 | 928 | 2187 |
| **VS-H→L** | 4.92 | 2.6 | 34.3 | 38.5 | 2.7 | 96 | 962 | 1193 |
| **VS-H→L** | 4.58 | 2.3 | 33.0 | 38.0 | 2.6 | 82 | 619 | 1674 |
| **VS-L** | 4.59 | 11.2 | 145.7 | 119.7 | 26.1 | 117 | 4828 | 17686 |
| **VS-L** | 4.48 | 10.1 | 117.1 | 89.0 | 16.7 | 116 | 1733 | 10575 |
| **VS-L** | 4.46 | 7.0 | 82.2 | 101.8 | 14.8 | 136 | 1129 | 7346 |

|  | Hydrolytic enzymatic activity (nmol g^-1^ hour^-1^) | | | | | | Proportion in total hydrolytic activity | | | Microbial biomass (μg g^-1^) | | | Microbial activities | | |
| --- | --- | --- | --- | --- | --- | --- | --- | --- | --- | --- | --- | --- | --- | --- | --- |
| **origin** | BG | CEL | PHO | Leu | NAG | total | C- mining | P-mining | N-mining | MB-C | MB-N | MB-P | respiration rate (μg C-CO_2_ g^-1^ hr^-1^) | amonification (ng N g^-1^ hr^-1^) | nitrification (ng N g^-1^ hr^-1^) |
| **FU-H** | 1633 | 521 | 1825 | 28.0 | 130.6 | 4138 | 0.52 | 0.04 | 0.44 | 4182 | 331 | 297 | 2.48 | 134.0 | 1165.2 |
| **FU-H** | 1663 | 460 | 1419 | 25.5 | 85.1 | 3652 | 0.58 | 0.03 | 0.39 | 2999 | 262 | 218 | 2.00 | 457.9 | 1390.3 |
| **FU-H** | 1110 | 264 | 823 | 36.3 | 97.3 | 2331 | 0.59 | 0.06 | 0.35 | 2845 | 242 | 193 | 1.65 | 15.2 | 843.9 |
| **FU-H→L** | 1560 | 318 | 2091 | 104.8 | 167.1 | 4240 | 0.44 | 0.06 | 0.49 | 2681 | 135 | 223 | 2.66 | 2253.4 | 1311.2 |
| **FU-H→L** | 1695 | 507 | 1946 | 20.7 | 127.9 | 4297 | 0.51 | 0.03 | 0.45 | 3630 | 316 | 254 | 2.04 | -25.6 | 1360.2 |
| **FU-H→L** | 962 | 298 | 1091 | 13.9 | 83.1 | 2448 | 0.51 | 0.04 | 0.45 | 2566 | 251 | 179 | 1.29 | 389.8 | 1197.2 |
| **FU-L** | 1073 | 272 | 893 | 61.6 | 106.1 | 2405 | 0.56 | 0.07 | 0.37 | 3482 | 316 | 211 | 1.08 | -3.5 | 54.1 |
| **FU-L** | 1115 | 275 | 865 | 43.5 | 86.4 | 2385 | 0.58 | 0.05 | 0.36 | 3684 | 331 | 239 | 0.82 | 26.1 | 48.7 |
| **FU-L** | 1158 | 278 | 837 | 25.3 | 66.6 | 2365 | 0.61 | 0.04 | 0.35 | 3886 | 346 | 266 | 0.55 | 55.8 | 43.3 |
| **VS-H** | 1156 | 267 | 1925 | 32.8 | 112.1 | 3494 | 0.41 | 0.04 | 0.55 | 1924 | 168 | 64 | 0.53 | 94.9 | 92.2 |
| **VS-H** | 494 | 102 | 1194 | 17.7 | 64.7 | 1872 | 0.32 | 0.04 | 0.64 | 1080 | 115 | 70 | 0.77 | 0.4 | 333.0 |
| **VS-H** | 730 | 134 | 1541 | 18.7 | 78.8 | 2503 | 0.35 | 0.04 | 0.62 | 1142 | 121 | 42 | 0.55 | 0.7 | 217.7 |
| **VS-H→L** | 492 | 86 | 1342 | 21.8 | 76.6 | 2019 | 0.29 | 0.05 | 0.66 | 1070 | 110 | 39 | 0.46 | 5.3 | 311.2 |
| **VS-H→L** | 441 | 75 | 1230 | 33.4 | 69.6 | 1849 | 0.28 | 0.06 | 0.67 | 936 | 108 | 36 | 2.81 | 65.5 | 172.7 |
| **VS-H→L** | 442 | 76 | 1035 | 19.3 | 49.5 | 1622 | 0.32 | 0.04 | 0.64 | 865 | 92 | 48 | 1.47 | 0.7 | 241.4 |
| **VS-L** | 2466 | 718 | 3717 | 161.9 | 147.8 | 7210 | 0.44 | 0.04 | 0.52 | 4106 | 452 | 376 | 1.41 | 126.9 | 2014.0 |
| **VS-L** | 1815 | 545 | 1636 | 28.9 | 83.6 | 4109 | 0.57 | 0.03 | 0.40 | 3462 | 432 | 346 | 1.40 | 15.8 | 1332.6 |
| **VS-L** | 889 | 246 | 967 | 23.7 | 50.3 | 2176 | 0.52 | 0.03 | 0.44 | 3192 | 357 | 280 | 1.28 | 40.0 | 1149.5 |

|  | P leaching  (mg P m^-2^ yr^-1^) | | NH4-N leaching (mg N m^-2^ yr^-1^) | | NO3-N leaching  (mg N m^-2^ yr^-1^) | | Aboveground biomass | | | | | | Root biomass | | | | | |
| --- | --- | --- | --- | --- | --- | --- | --- | --- | --- | --- | --- | --- | --- | --- | --- | --- | --- | --- |
| **origin** | 2014 | 2015 | 2014 | 2015 | 2014 | 2015 | %N | %C | %P | C/N | C/P | N/P | %N | %C | %P | C/N | C/P | N/P |
| **FU-H** | 5.7 | 22.8 | 117.3 | 88.2 | 394.0 | 46.0 | 1.3 | 45.4 | 0.1 | 40.1 | 1752 | 43.7 | 1.0 | 40.8 | 0.1 | 46.3 | 1224 | 26.4 |
| **FU-H** | 10.2 | 22.4 | 82.4 | 76.9 | 624.3 | 251.0 | 2.1 | 45.0 | 0.2 | 25.1 | 733 | 29.2 | 1.2 | 41.8 | 0.1 | 40.8 | 1367 | 33.5 |
| **FU-H** | 17.6 | 15.9 | 260.7 | 79.2 | 606.9 | 62.4 | 1.0 | 45.8 | 0.1 | 52.5 | 1990 | 37.9 | 0.7 | 33.7 | 0.1 | 55.3 | 1437 | 26.0 |
| **FU-H→L** | 26.4 | 29.6 | 1112.6 | 671.8 | 1378.6 | 391.3 | 3.6 | 46.0 | 0.3 | 14.9 | 452 | 30.3 | 2.2 | 43.9 | 0.1 | 23.3 | 949 | 40.9 |
| **FU-H→L** | 41.7 | 36.6 | 2060.4 | 100.4 | 2095.3 | 306.7 | 1.5 | 45.7 | 0.1 | 36.6 | 953 | 26.1 | 1.0 | 43.5 | 0.1 | 50.8 | 1279 | 25.2 |
| **FU-H→L** | 19.3 | 20.3 | 181.6 | 292.4 | 475.3 | 920.8 | 2.4 | 45.6 | 0.2 | 22.3 | 614 | 27.6 | 1.3 | 44.4 | 0.1 | 39.4 | 986 | 25.0 |
| **FU-L** | 33.0 | 50.6 | 560.7 | 246.0 | 35.1 | 132.1 | 1.2 | 45.5 | 0.1 | 44.0 | 794 | 18.1 | 1.0 | 42.6 | 0.2 | 49.5 | 693 | 14.0 |
| **FU-L** | 19.8 | 44.1 | 312.2 | 161.1 | 24.5 | 90.6 | 1.1 | 45.3 | 0.1 | 46.4 | 818 | 17.6 | 0.8 | 42.2 | 0.1 | 65.5 | 907 | 13.9 |
| **FU-L** | 6.6 | 37.6 | 63.7 | 76.2 | 14.0 | 49.0 | 1.1 | 45.1 | 0.1 | 48.9 | 841 | 17.2 | 0.6 | 41.8 | 0.1 | 81.6 | 1122 | 13.8 |
| **VS-H** | 2.1 | 30.6 | 68.6 | 135.6 | 173.2 | 73.7 | 1.5 | 45.1 | 0.1 | 33.9 | 1561 | 46.0 | 0.9 | 36.0 | 0.1 | 47.1 | 1725 | 36.7 |
| **VS-H** | 12.6 | 17.2 | 308.7 | 63.4 | 809.5 | 808.9 | 1.6 | 42.1 | 0.0 | 31.6 | 2797 | 88.6 | 1.0 | 32.4 | 0.0 | 38.5 | 1987 | 51.6 |
| **VS-H** | 5.9 | 14.8 | 150.5 | 56.7 | 618.1 | 205.8 | 1.5 | 44.8 | 0.0 | 35.4 | 2648 | 74.8 | 0.8 | 26.4 | 0.1 | 37.4 | 1265 | 33.9 |
| **VS-H→L** | 10.1 | 38.6 | 144.8 | 107.1 | 295.1 | 309.9 | 1.8 | 37.3 | 0.1 | 24.7 | 1031 | 41.7 | 0.8 | 28.1 | 0.0 | 43.4 | 1552 | 35.8 |
| **VS-H→L** | 9.8 | 32.8 | 825.4 | 261.2 | 1900.5 | 370.4 | 2.0 | 43.5 | 0.1 | 26.0 | 1203 | 46.3 | 1.0 | 32.8 | 0.1 | 39.1 | 1315 | 33.6 |
| **VS-H→L** | 9.9 | 23.0 | 485.1 | 301.2 | 1097.8 | 1313.6 | 1.6 | 44.7 | 0.1 | 33.2 | 1589 | 47.8 | 0.7 | 20.9 | 0.1 | 36.4 | 1075 | 29.5 |
| **VS-L** | 19.9 | 33.9 | 568.2 | 373.1 | 630.6 | 1063.9 | 3.1 | 43.0 | 0.1 | 16.3 | 850 | 52.3 | 1.2 | 42.1 | 0.0 | 41.9 | 2214 | 52.9 |
| **VS-L** | 9.8 | 19.4 | 435.8 | 128.1 | 2494.9 | 1187.0 | 3.2 | 42.6 | 0.1 | 15.7 | 857 | 54.5 | 1.4 | 42.7 | 0.1 | 36.8 | 1612 | 43.8 |
| **VS-L** | 18.3 | 27.3 | 170.3 | 137.4 | 770.4 | 706.8 | 2.3 | 42.7 | 0.1 | 21.3 | 974 | 45.7 | 1.2 | 43.3 | 0.1 | 41.3 | 1359 | 32.9 |
