# Supplementary material for: Soil warming during winter period enhanced soil N and P availability and leaching in alpine grasslands: A transplant study
Source: PLoS One. 2022 Aug 2;17(8):e0272143. doi: 10.1371/journal.pone.0272143 (PMC9345486; doi:10.1371/journal.pone.0272143)
Supplement: S2 Table — Mean (standard deviations, n = 3) are shown. Characteristics where data are given in bold differ significantly between mesocosm and free soils (p < 0.05). (DOCX) [file pone.0272143.s002.docx]

**Table S2. Comparison of physico-chemical and biochemical characteristics of mesocosm and ambient soils sampled at the end of the 2-year experiment in September 2015**. Mean (standard deviations, n = 3) are shown. Characteristics where data are given in bold differ significantly between mesocosm and free soils (*p* < 0.05).

|  |  | **FU-H** | | **VS-H** | |
| --- | --- | --- | --- | --- | --- |
| Soil property | Units | Mesocosm soil | Ambient soil | Mesocosm soil | Ambient soil |
| pH_(H2O)_ |  | 4.41 (0.32) | 4.69 (0.36) | 4.82 (0.18) | 4.51 (0.07) |
| CEC | meq kg^-1^ | 94 (14) | 104 (5.2) | 123 (19) | 144 (7.2) |
| BS | % | 17 (4.5) | 13 (0.63) | 5.7 (2.1) | 8.7 (2.2) |
| K^+^ | meq kg^-1^ | 4.4 (0.82) | 3.8 (0.19) | 5.4 (0.98) | 4.6 (0.23) |
| Na^+^ | meq kg^-1^ | 0.53 (0.34) | 0.28 (0.01) | 0.44 (0.01) | 0.37 (0.02) |
| Ca^2+^ | meq kg^-1^ | 6.6 (2.3) | 5.9 (0.30) | 4.5 (1.4) | 5.6 (0.28) |
| Mg^2+^ | meq kg^-1^ | 3.3 (0.77) | 3.0 (0.15) | 4.3 (0.17) | 1.9 (0.10) |
| Al^3+^ | meq kg^-1^ | 53 (11) | 63 (9.5) | 104 (3.6) | 110 (17) |
| H^+^ | meq kg^-1^ | 26 (1.8) | 28 (1.4) | 28 (0.24) | 22 (4.4) |
| C | mg kg^-1^ | 90 (32) | 104 (27) | 45 (19) | 36 (15) |
| N | mg kg^-1^ | 6.7 (2.2) | 6.5 (1.7) | 3.2 (1.5) | 2.6 (1.2) |
| P | mg kg^-1^ | 0.93 (0.19) | 0.97 (0.23) | 0.45 (0.12) | 0.47 (0.09) |
| DOC | mg kg^-1^ | **96 (3.3)** | **147 (35)** | 42 (10) | 60 (2.2) |
| DN | mg kg^-1^ | 6.4 (1.5) | 15 (1.5) | **3.7 (1.3)** | **10 (1.8)** |
| NH_4_-N | mg kg^-1^ | 1.1 (0.52) | 2.9 (1.2) | 0.59 (0.10) | 0.43 (0.10) |
| NO_3_-N | mg kg^-1^ | 3.0 (1.4) | 3.4 (0.13) | 2.4 (1.08) | 1.5 (0.14) |
| SRP | mg kg^-1^ | 0.26 (0.07) | 0.32 (0.09) | 0.07 (0.01) | 0.16 (0.10) |
| MB-C | mg kg^-1^ | 3.3 (0.73) | 2.6 (0.13) | 1.1 (0.10) | 2.0 (0.77) |
| MB-N | mg kg^-1^ | 0.28 (0.05) | 0.32 (0.02) | 0.11 (0.01) | 0.14 (0.08) |
| MB-P | mg kg^-1^ | 0.24 (0.05) | 0.20 (0.04) | 0.049 (0.01) | 0.043 (0.01) |
