# Supplementary material for: Soil warming during winter period enhanced soil N and P availability and leaching in alpine grasslands: A transplant study
Source: PLoS One. 2022 Aug 2;17(8):e0272143. doi: 10.1371/journal.pone.0272143 (PMC9345486; doi:10.1371/journal.pone.0272143)
Supplement: S3 Table — (DOCX) [file pone.0272143.s003.docx]

**Table S3**. Effect of valley, downward transfer and their interaction on mesocosm soil bacterial community composition – results of PERMANOVA.

| **Parameter** | **Df** | **SS** | **F** | **R^2^** | ***p*** |
| --- | --- | --- | --- | --- | --- |
| Valley | 1 | 0.385 | 5.52 | 0.363 | <0.001 |
| Transfer | 1 | 0.092 | 1.31 | 0.086 | 0.193 |
| Valley:Transfer | 1 | 0.094 | 1.35 | 0.089 | 0.175 |
