# Supplementary material for: Soil warming during winter period enhanced soil N and P availability and leaching in alpine grasslands: A transplant study
Source: PLoS One. 2022 Aug 2;17(8):e0272143. doi: 10.1371/journal.pone.0272143 (PMC9345486; doi:10.1371/journal.pone.0272143)
Supplement: S4 Table — Numbers represent mean (relative abundance in % in case of orders) with standard deviation in brackets. χ2 and p-value for test of differences between valleys are presented. Small letters denote significant differences at p < 0.05. (DOCX) [file pone.0272143.s004.docx]

**Table S4**. **The most abundant bacterial orders (> 1% in at least one variant) and alpha diversity measures in *in situ* and transferred mesocosms.** Numbers represent mean (relative abundance in % in case of orders) with standard deviation in brackets. χ2 and p-value for test of differences between valleys are presented. Small letters denote significant differences at *p* < 0.05.

| **Order (Class)** |  | **FU-H** | **FU-H→L** | **VS-H** | **VS-H→L** | χ **^2^** | ***p*** |
| --- | --- | --- | --- | --- | --- | --- | --- |
| Acetobacterales | (Alphaproteobacteria) | 1.62 (0.85) | 1.19 (0.1) | 0.97 (0.15) | 0.91 (0.13) | n.s. | |
| Acidobacteriales | (Acidobacteriae) | 13.1 (0.7) b | 12.9 (1.5) b | 15.4 (1.1) ab | 16.3 (2.2) a | 10.2 | 0.001 |
| Armatimonadales | (Armatimonadia) | 1.24 (0.17) a | 0.67 (0.32) b | 0.65 (0.06) b | 0.33 (0.03) b | 15.7 | <0.001 |
| Bryobacterales | (Acidobacteriae) | 2.3 (0.23) b | 2.24 (0.36) b | 2.38 (0.33) ab | 3.06 (0.23) a | 7.4 | 0.007 |
| Burkholderiales | (Gammaproteobacteria) | 2.48 (0.85) a | 4.94 (3.26) a | 1.73 (0.33) a | 1.78 (0.63) a | 5.6 | 0.018 |
| Caulobacterales | (Alphaproteobacteria) | 2.13 (0.36) a | 1.57 (0.19) b | 0.55 (0.13) c | 0.69 (0.14) c | 86.5 | <0.001 |
| Chitinophagales | (Bacteroidia) | 4.09 (0.42) a | 4.07 (0.57) a | 1.89 (0.06) b | 2.01 (0.2) b | 98.8 | <0.001 |
| Chthoniobacterales | (Verrucomicrobiae) | 2.82 (0.46) b | 2.57 (0.27) b | 7.38 (1.63) a | 6.08 (1.13) a | 67.0 | <0.001 |
| Frankiales | (Actinobacteria) | 4.16 (0.54) a | 3.34 (1.03) a | 4.83 (0.82) a | 4.85 (0.57) a | 5.5 | 0.019 |
| Gemmatales | (Planctomycetes) | 4.49 (0.31) ab | 3.33 (1.48) b | 6.75 (0.19) a | 6.41 (0.5) a | 19.3 | <0.001 |
| Isosphaerales | (Planctomycetes) | 1.45 (0.23) | 1.08 (0.32) | 1.51 (0.31) | 1.4 (0.22) | n.s. | |
| Ktedonobacterales | (Ktedonobacteria) | 10.65 (0.76) a | 6.12 (2.83) b | 5.18 (0.38) b | 5.69 (1.43) b | 5.7 | 0.017 |
| Micropepsales | (Alphaproteobacteria) | 1.88 (0.22) | 3.3 (1.58) | 1.97 (0.26) | 1.72 (0.01) | n.s. | |
| Pedosphaerales | (Verrucomicrobiae) | 1.84 (0.59) ab | 2.34 (0.26) a | 1.38 (0.1) b | 1.49 (0.23) b | 9.4 | 0.002 |
| Polyangiales | (Polyangia) | 1.07 (0.14) | 0.84 (0.43) | 0.93 (0.26) | 0.86 (0.17) | n.s. | |
| RCP2-54 | (RCP2-54) | 0.46 (0.2) b | 0.51 (0.17) b | 1.18 (0.2) a | 1.68 (0.15) a | 54.5 | <0.001 |
| Rhizobiales | (Alphaproteobacteria) | 6.91 (1.01) a | 7.1 (1.24) a | 8.71 (0.24) a | 8.52 (1.08) a | 6.1 | 0.013 |
| Solibacterales | (Acidobacteriae) | 2.56 (0.28) c | 2.25 (0.5) c | 5.03 (0.68) a | 3.75 (0.33) b | 49.4 | <0.001 |
| Solirubrobacterales | (Thermoleophilia) | 4.32 (1.07) | 3.04 (1.44) | 3.68 (0.57) | 2.67 (0.51) | n.s | |
| Sphingobacteriales | (Bacteroidia) | 2.34 (0.19) a | 5.68 (5.88) a | 0.87 (0.12) a | 0.96 (0.34) a | 6.7 | 0.010 |
| Subgroup 2 | (Acidobacteriae) | 5.03 (0.71) b | 5.06 (1.39) b | 7.57 (0.77) a | 10.41 (0.82) a | 39.4 | <0.001 |
| Tepidisphaerales | (Phycisphaerae) | 0.9 (0.18) | 0.83 (0.1) | 0.92 (0.02) | 1.06 (0.28) | n.s. | |
| WD260 | (Gammaproteobacteria) | 2.73 (0.12) a | 2.63 (0.92) a | 1.76 (0.42) ab | 1.54 (0.05) b | 12.4 | <0.001 |
| WPS-2 | (WPS-2) | 4.03 (0.84) a | 2.4 (1.02) b | 2.92 (0.1) ab | 1.69 (0.11) b | 4.5 | 0.033 |
| Xanthomonadales | (Gammaproteobacteria) | 1.22 (0.05) ab | 1.92 (1.15) a | 0.45 (0.23) b | 0.55 (0.04) b | 15.0 | <0.001 |
|  |  |  |  |  |  |  |  |
| OTU richness |  | 1487 (174) | 1479 (115) | 1373 (278) | 1477 (95) | n.s. | |
| Chao1 |  | 1784 (264) | 1768 (97) | 1620 (421) | 1786 (123) | n.s. | |
| Shannon |  | 6.52 (0.13) | 6.54 (0.16) | 6.31 (0.23) | 6.38 (0.11) | n.s. | |
